# Supplementary material for: Logistic Mixed‐Effects Model Analysis With Pseudo‐Observations for Estimating Risk Ratios in Clustered Binary Data Analysis
Source: Stat Med. 2025 Sep 22;44(20-22):e70280. doi: 10.1002/sim.70280 (PMC12454234; doi:10.1002/sim.70280)
Supplement: Supplementary file 1 — Data S1: sim70280‐sup‐0001‐Supinfo.zip. [file SIM-44-0-s001.zip › sim70280-sup-0003-Supinfo3@SupportingInformation20250801.pdf]

SUPPORTING INFORMATION for

Logistic mixed-effects model analysis with pseudo-observations for estimating risk ratios in clustered binary data analysis

Hisashi Noma[1,2] and Masahiko Gosho[3]

[1] *Department of Interdisciplinary Statistical Mathematics, The Institute of Statistical Mathematics, Tokyo, Japan*

[2] *The Graduate Institute for Advanced Studies, The Graduate University for Advanced Studies, Tokyo, Japan*

[3] *Department of Biostatistics, Institute of Medicine, University of Tsukuba, Tsukuba, Japan*

e-Appendix A: Supplementary data for the simulation studies

As supplementary data of the simulations studies in Section 4, we present the simulation results for the estimation of regression coefficients $\beta_0, \beta_2, \ldots, \beta_5$ in e-Tables 1-10. For the evaluation measures, we used the means and standard deviations of the odds ratio and risk ratio estimates from the 1,000 simulations. Also, for the proposed method, we calculated mean of the bootstrap standard error estimates and root mean squared error (RMSE) of the regression coefficient estimates, and the coverage rate and expected width of the 95% confidence intervals.

e-Table 1. Results of the simulation studies for the regression coefficient estimators of β_0 (N = 50, 100) [†].

				Linear mixed-effects model (log odds ratio)		Pseudo-observations approach (log risk ratio)					
N	P_{event}	τ	β_0	Mean	SE	Mean	SE	$\widehat{SE}$	RMSE	CR	EW
50	0.20	0.02	−2.32	−2.422	1.314	−2.428	1.014	1.512	1.019	0.921	5.204
50	0.20	0.02	−2.43	−2.584	1.361	−2.554	1.100	1.445	1.107	0.948	4.906
50	0.20	0.05	−2.32	−2.411	1.231	−2.414	0.914	1.459	0.919	0.927	5.052
50	0.20	0.05	−2.43	−2.575	1.276	−2.533	0.930	1.506	0.935	0.942	5.041
50	0.20	0.10	−2.32	−2.430	1.281	−2.431	0.971	1.505	0.977	0.942	5.223
50	0.20	0.10	−2.43	−2.551	1.259	−2.511	0.923	1.471	0.926	0.938	4.950
50	0.40	0.02	−1.63	−1.647	1.071	−1.668	0.534	0.643	0.535	0.943	2.403
50	0.40	0.02	−1.73	−1.871	1.156	−1.748	0.530	0.623	0.530	0.940	2.343
50	0.40	0.05	−1.63	−1.631	1.095	−1.657	0.547	0.647	0.547	0.937	2.438
50	0.40	0.05	−1.73	−1.878	1.121	−1.750	0.490	0.617	0.490	0.954	2.306
50	0.40	0.10	−1.63	−1.688	1.028	−1.678	0.513	0.635	0.515	0.941	2.353
50	0.40	0.10	−1.73	−1.894	1.141	−1.770	0.509	0.624	0.511	0.948	2.328
100	0.20	0.02	−2.32	−2.293	0.767	−2.323	0.573	0.611	0.573	0.946	2.371
100	0.20	0.02	−2.43	−2.493	0.802	−2.467	0.581	0.606	0.582	0.933	2.332
100	0.20	0.05	−2.32	−2.405	0.765	−2.407	0.568	0.619	0.575	0.946	2.384
100	0.20	0.05	−2.43	−2.497	0.774	−2.467	0.555	0.604	0.555	0.948	2.342
100	0.20	0.10	−2.32	−2.357	0.815	−2.369	0.607	0.627	0.609	0.936	2.419
100	0.20	0.10	−2.43	−2.517	0.781	−2.475	0.563	0.605	0.564	0.946	2.340
100	0.40	0.02	−1.63	−1.612	0.688	−1.631	0.350	0.360	0.350	0.945	1.413
100	0.40	0.02	−1.73	−1.872	0.728	−1.743	0.333	0.348	0.333	0.951	1.363
100	0.40	0.05	−1.63	−1.578	0.699	−1.618	0.349	0.359	0.349	0.945	1.408
100	0.40	0.05	−1.73	−1.819	0.763	−1.724	0.350	0.350	0.350	0.934	1.371
100	0.40	0.10	−1.63	−1.612	0.695	−1.632	0.347	0.362	0.347	0.940	1.419
100	0.40	0.10	−1.73	−1.838	0.759	−1.729	0.345	0.354	0.344	0.945	1.386

[†] Mean, SE: Mean and standard deviation of the estimates, $\widehat{SE}$: Mean of the standard error estimates, RMSE: Root mean squared error of the estimates, CR, EW: Coverage rate and expected width of the 95% confidence intervals.

e-Table 2. Results of the simulation studies for the regression coefficient estimators of β_0 (N = 200, 500)[†].

				Linear mixed-effects model (log odds ratio)		Pseudo-observations approach (log risk ratio)					
N	P_{event}	τ	β_0	Mean	SE	Mean	SE	$\widehat{SE}$	RMSE	CR	EW
200	0.20	0.02	−2.32	−2.352	0.539	−2.365	0.400	0.402	0.403	0.933	1.572
200	0.20	0.02	−2.43	−2.476	0.528	−2.451	0.385	0.396	0.386	0.948	1.550
200	0.20	0.05	−2.32	−2.312	0.532	−2.365	0.400	0.402	0.403	0.933	1.572
200	0.20	0.05	−2.43	−2.500	0.552	−2.451	0.385	0.396	0.386	0.948	1.550
200	0.20	0.10	−2.32	−2.349	0.493	−2.336	0.396	0.404	0.397	0.947	1.579
200	0.20	0.10	−2.43	−2.447	0.538	−2.471	0.400	0.398	0.402	0.936	1.557
200	0.40	0.02	−1.63	−1.614	0.474	−2.360	0.368	0.403	0.370	0.956	1.578
200	0.40	0.02	−1.73	−1.847	0.506	−2.432	0.389	0.395	0.389	0.945	1.546
200	0.40	0.05	−1.63	−1.639	0.473	−1.637	0.239	0.243	0.239	0.950	0.950
200	0.40	0.05	−1.73	−1.860	0.519	−1.736	0.233	0.234	0.233	0.937	0.915
200	0.40	0.10	−1.63	−1.634	0.495	−1.649	0.242	0.245	0.243	0.947	0.956
200	0.40	0.10	−1.73	−1.837	0.496	−1.739	0.236	0.235	0.236	0.944	0.918
500	0.20	0.02	−2.32	−2.312	0.341	−1.642	0.246	0.245	0.246	0.938	0.957
500	0.20	0.02	−2.43	−2.452	0.327	−1.725	0.228	0.237	0.228	0.945	0.926
500	0.20	0.05	−2.32	−2.295	0.327	−2.339	0.257	0.248	0.258	0.932	0.968
500	0.20	0.05	−2.43	−2.438	0.337	−2.442	0.237	0.243	0.237	0.949	0.949
500	0.20	0.10	−2.32	−2.308	0.339	−2.328	0.243	0.248	0.243	0.956	0.968
500	0.20	0.10	−2.43	−2.452	0.335	−2.431	0.245	0.242	0.245	0.940	0.947
500	0.40	0.02	−1.63	−1.627	0.288	−2.331	0.252	0.247	0.252	0.934	0.967
500	0.40	0.02	−1.73	−1.822	0.325	−2.435	0.243	0.243	0.243	0.953	0.949
500	0.40	0.05	−1.63	−1.615	0.293	−1.640	0.145	0.150	0.145	0.956	0.586
500	0.40	0.05	−1.73	−1.842	0.322	−1.719	0.150	0.145	0.150	0.940	0.564
500	0.40	0.10	−1.63	−1.619	0.309	−1.636	0.148	0.150	0.148	0.951	0.586
500	0.40	0.10	−1.73	−1.835	0.318	−1.725	0.148	0.145	0.148	0.947	0.567

[†] Mean, SE: Mean and standard deviation of the estimates, $\widehat{SE}$: Mean of the standard error estimates, RMSE: Root mean squared error of the estimates, CR, EW: Coverage rate and expected width of the 95% confidence intervals.

e-Table 3. Results of the simulation studies for the regression coefficient estimators of β_2 (= 0.71; N = 50, 100)[†].

			Linear mixed-effects model (log odds ratio)		Pseudo-observations approach (log risk ratio)					
N	P_{event}	τ	Mean	SE	Mean	SE	$\widehat{SE}$	RMSE	CR	EW
50	0.20	0.02	0.992	0.471	0.742	0.360	0.459	0.361	0.944	1.567
50	0.20	0.02	1.022	0.457	0.745	0.343	0.462	0.345	0.945	1.522
50	0.20	0.05	0.990	0.457	0.738	0.351	0.448	0.352	0.940	1.531
50	0.20	0.05	1.020	0.462	0.742	0.348	0.454	0.349	0.945	1.540
50	0.20	0.10	0.983	0.473	0.731	0.364	0.461	0.364	0.928	1.557
50	0.20	0.10	1.033	0.454	0.747	0.341	0.463	0.343	0.950	1.545
50	0.40	0.02	1.470	0.413	0.728	0.209	0.223	0.210	0.948	0.872
50	0.40	0.02	1.653	0.436	0.726	0.205	0.220	0.205	0.948	0.857
50	0.40	0.05	1.485	0.418	0.740	0.216	0.223	0.217	0.943	0.873
50	0.40	0.05	1.651	0.448	0.725	0.207	0.218	0.207	0.937	0.851
50	0.40	0.10	1.462	0.412	0.715	0.208	0.225	0.207	0.945	0.878
50	0.40	0.10	1.644	0.451	0.726	0.206	0.221	0.206	0.953	0.866
100	0.20	0.02	0.958	0.313	0.720	0.240	0.239	0.240	0.932	0.935
100	0.20	0.02	0.987	0.308	0.722	0.229	0.235	0.229	0.936	0.921
100	0.20	0.05	0.953	0.306	0.718	0.238	0.241	0.238	0.932	0.946
100	0.20	0.05	1.003	0.318	0.732	0.237	0.235	0.237	0.936	0.922
100	0.20	0.10	0.970	0.311	0.728	0.237	0.242	0.238	0.944	0.948
100	0.20	0.10	0.973	0.303	0.707	0.225	0.235	0.225	0.952	0.924
100	0.40	0.02	1.426	0.267	0.712	0.139	0.143	0.139	0.951	0.560
100	0.40	0.02	1.605	0.295	0.718	0.141	0.139	0.141	0.937	0.542
100	0.40	0.05	1.430	0.260	0.718	0.137	0.143	0.137	0.957	0.562
100	0.40	0.05	1.589	0.288	0.712	0.136	0.139	0.136	0.943	0.545
100	0.40	0.10	1.436	0.275	0.715	0.141	0.144	0.141	0.955	0.564
100	0.40	0.10	1.606	0.293	0.718	0.137	0.141	0.137	0.952	0.552

[†] Mean, SE: Mean and standard deviation of the estimates, $\widehat{SE}$: Mean of the standard error estimates, RMSE: Root mean squared error of the estimates, CR, EW: Coverage rate and expected width of the 95% confidence intervals.

e-Table 4. Results of the simulation studies for the regression coefficient estimators of β_2 (= 0.71; N = 200, 500)[†].

			Linear mixed-effects model (log odds ratio)		Pseudo-observations approach (log risk ratio)					
N	P_{event}	τ	Mean	SE	Mean	SE	$\widehat{SE}$	RMSE	CR	EW
200	0.20	0.02	0.941	0.205	0.712	0.159	0.162	0.159	0.954	0.633
200	0.20	0.02	0.971	0.206	0.714	0.158	0.159	0.158	0.946	0.622
200	0.20	0.05	0.944	0.204	0.714	0.158	0.163	0.158	0.943	0.639
200	0.20	0.05	0.975	0.204	0.716	0.155	0.160	0.155	0.955	0.624
200	0.20	0.10	0.954	0.211	0.719	0.162	0.163	0.162	0.945	0.636
200	0.20	0.10	0.986	0.211	0.722	0.160	0.159	0.160	0.942	0.623
200	0.40	0.02	1.425	0.184	0.719	0.096	0.099	0.096	0.946	0.386
200	0.40	0.02	1.597	0.204	0.719	0.097	0.095	0.097	0.940	0.372
200	0.40	0.05	1.424	0.189	0.717	0.098	0.098	0.098	0.946	0.385
200	0.40	0.05	1.577	0.206	0.708	0.096	0.095	0.096	0.941	0.370
200	0.40	0.10	1.427	0.185	0.713	0.094	0.099	0.094	0.954	0.386
200	0.40	0.10	1.580	0.203	0.709	0.095	0.096	0.095	0.936	0.374
500	0.20	0.02	0.942	0.131	0.716	0.102	0.101	0.102	0.938	0.392
500	0.20	0.02	0.967	0.126	0.715	0.097	0.099	0.097	0.953	0.385
500	0.20	0.05	0.945	0.133	0.718	0.104	0.101	0.104	0.940	0.393
500	0.20	0.05	0.970	0.126	0.715	0.096	0.098	0.096	0.956	0.384
500	0.20	0.10	0.935	0.127	0.709	0.098	0.101	0.098	0.955	0.393
500	0.20	0.10	0.979	0.128	0.720	0.097	0.099	0.097	0.953	0.385
500	0.40	0.02	1.415	0.116	0.716	0.061	0.061	0.061	0.948	0.239
500	0.40	0.02	1.575	0.127	0.711	0.060	0.059	0.060	0.943	0.230
500	0.40	0.05	1.411	0.121	0.712	0.064	0.061	0.064	0.936	0.239
500	0.40	0.05	1.575	0.128	0.710	0.060	0.059	0.060	0.945	0.231
500	0.40	0.10	1.422	0.118	0.714	0.063	0.062	0.063	0.943	0.240
500	0.40	0.10	1.565	0.122	0.705	0.057	0.059	0.058	0.952	0.232

[†] Mean, SE: Mean and standard deviation of the estimates, $\widehat{SE}$: Mean of the standard error estimates, RMSE: Root mean squared error of the estimates, CR, EW: Coverage rate and expected width of the 95% confidence intervals.

e-Table 5. Results of the simulation studies for the regression coefficient estimators of β_3 (= 0.28; N = 50, 100)[†].

			Linear mixed-effects model (log odds ratio)		Pseudo-observations approach (log risk ratio)					
N	P_{event}	τ	Mean	SE	Mean	SE	$\widehat{SE}$	RMSE	CR	EW
50	0.20	0.02	0.415	0.485	0.299	0.360	0.429	0.360	0.936	1.552
50	0.20	0.02	0.405	0.469	0.279	0.340	0.437	0.340	0.945	1.541
50	0.20	0.05	0.412	0.456	0.297	0.341	0.431	0.342	0.954	1.555
50	0.20	0.05	0.405	0.494	0.284	0.361	0.454	0.361	0.937	1.547
50	0.20	0.10	0.396	0.490	0.285	0.364	0.450	0.364	0.929	1.614
50	0.20	0.10	0.399	0.469	0.275	0.343	0.457	0.343	0.953	1.568
50	0.40	0.02	0.615	0.427	0.281	0.209	0.222	0.209	0.933	0.868
50	0.40	0.02	0.690	0.454	0.280	0.209	0.219	0.209	0.937	0.857
50	0.40	0.05	0.604	0.440	0.274	0.221	0.222	0.221	0.926	0.871
50	0.40	0.05	0.660	0.441	0.270	0.197	0.218	0.197	0.953	0.852
50	0.40	0.10	0.641	0.420	0.291	0.208	0.226	0.208	0.940	0.881
50	0.40	0.10	0.655	0.459	0.264	0.209	0.222	0.210	0.934	0.871
100	0.20	0.02	0.384	0.304	0.279	0.227	0.238	0.227	0.938	0.932
100	0.20	0.02	0.402	0.316	0.281	0.229	0.234	0.229	0.935	0.918
100	0.20	0.05	0.422	0.293	0.306	0.219	0.239	0.221	0.944	0.936
100	0.20	0.05	0.387	0.323	0.271	0.235	0.234	0.235	0.947	0.920
100	0.20	0.10	0.389	0.326	0.281	0.243	0.241	0.243	0.932	0.944
100	0.20	0.10	0.408	0.300	0.284	0.217	0.234	0.217	0.960	0.917
100	0.40	0.02	0.612	0.271	0.281	0.136	0.141	0.136	0.950	0.554
100	0.40	0.02	0.667	0.282	0.276	0.131	0.137	0.131	0.951	0.536
100	0.40	0.05	0.595	0.275	0.274	0.138	0.142	0.138	0.948	0.555
100	0.40	0.05	0.644	0.291	0.267	0.134	0.138	0.135	0.939	0.540
100	0.40	0.10	0.609	0.274	0.279	0.137	0.142	0.137	0.953	0.557
100	0.40	0.10	0.636	0.304	0.266	0.141	0.140	0.142	0.937	0.547

[†] Mean, SE: Mean and standard deviation of the estimates, $\widehat{SE}$: Mean of the standard error estimates, RMSE: Root mean squared error of the estimates, CR, EW: Coverage rate and expected width of the 95% confidence intervals.

e-Table 6. Results of the simulation studies for the regression coefficient estimators of β_3 (= 0.28; N = 200, 500) †.

			Linear mixed-effects model (log odds ratio)		Pseudo-observations approach (log risk ratio)					
N	P_{event}	τ	Mean	SE	Mean	SE	$\widehat{SE}$	RMSE	CR	EW
200	0.20	0.02	0.393	0.213	0.286	0.159	0.160	0.159	0.949	0.627
200	0.20	0.02	0.403	0.213	0.283	0.156	0.158	0.156	0.949	0.618
200	0.20	0.05	0.389	0.214	0.283	0.160	0.162	0.160	0.945	0.633
200	0.20	0.05	0.402	0.218	0.282	0.159	0.158	0.159	0.938	0.619
200	0.20	0.10	0.388	0.208	0.282	0.156	0.161	0.156	0.946	0.633
200	0.20	0.10	0.395	0.212	0.277	0.153	0.158	0.153	0.952	0.617
200	0.40	0.02	0.607	0.187	0.281	0.093	0.096	0.093	0.950	0.376
200	0.40	0.02	0.651	0.200	0.271	0.092	0.094	0.093	0.947	0.366
200	0.40	0.05	0.608	0.190	0.282	0.096	0.097	0.096	0.947	0.379
200	0.40	0.05	0.658	0.196	0.275	0.093	0.094	0.093	0.954	0.368
200	0.40	0.10	0.603	0.199	0.278	0.099	0.098	0.099	0.936	0.382
200	0.40	0.10	0.643	0.195	0.269	0.089	0.095	0.090	0.963	0.370
500	0.20	0.02	0.387	0.129	0.283	0.098	0.100	0.098	0.950	0.389
500	0.20	0.02	0.396	0.134	0.281	0.098	0.098	0.098	0.946	0.382
500	0.20	0.05	0.381	0.130	0.279	0.097	0.100	0.097	0.954	0.389
500	0.20	0.05	0.396	0.132	0.279	0.096	0.097	0.096	0.944	0.380
500	0.20	0.10	0.387	0.131	0.282	0.099	0.100	0.099	0.960	0.391
500	0.20	0.10	0.390	0.132	0.274	0.097	0.098	0.097	0.950	0.381
500	0.40	0.02	0.602	0.118	0.280	0.060	0.060	0.060	0.955	0.234
500	0.40	0.02	0.642	0.125	0.268	0.058	0.058	0.059	0.948	0.227
500	0.40	0.05	0.607	0.123	0.282	0.062	0.060	0.062	0.934	0.234
500	0.40	0.05	0.644	0.129	0.269	0.060	0.058	0.061	0.927	0.227
500	0.40	0.10	0.600	0.118	0.277	0.059	0.060	0.059	0.944	0.236
500	0.40	0.10	0.631	0.127	0.264	0.058	0.059	0.060	0.931	0.229

† Mean, SE: Mean and standard deviation of the estimates, $\widehat{SE}$: Mean of the standard error estimates, RMSE: Root mean squared error of the estimates, CR, EW: Coverage rate and expected width of the 95% confidence intervals.

e-Table 7. Results of the simulation studies for the regression coefficient estimators of β_4 (= −0.05; N = 50, 100) [†].

			Linear mixed-effects model (log odds ratio)		Pseudo-observations approach (log risk ratio)					
N	P_{event}	τ	Mean	SE	Mean	SE	$\widehat{SE}$	RMSE	CR	EW
50	0.20	0.02	−0.047	0.766	−0.018	0.638	1.006	0.639	0.939	3.177
50	0.20	0.02	−0.052	0.844	−0.013	0.757	0.878	0.758	0.963	2.737
50	0.20	0.05	−0.085	0.603	−0.051	0.437	0.934	0.437	0.948	3.007
50	0.20	0.05	−0.043	0.634	−0.019	0.449	0.915	0.449	0.933	2.920
50	0.20	0.10	−0.029	0.764	−0.006	0.614	0.988	0.615	0.944	3.123
50	0.20	0.10	−0.071	0.661	−0.034	0.479	0.900	0.479	0.940	2.807
50	0.40	0.02	−0.133	0.531	−0.038	0.247	0.366	0.248	0.945	1.239
50	0.40	0.02	−0.214	0.566	−0.056	0.234	0.343	0.234	0.941	1.172
50	0.40	0.05	−0.149	0.530	−0.045	0.246	0.373	0.246	0.945	1.292
50	0.40	0.05	−0.180	0.566	−0.042	0.222	0.344	0.222	0.951	1.163
50	0.40	0.10	−0.125	0.530	−0.038	0.247	0.362	0.247	0.947	1.205
50	0.40	0.10	−0.185	0.613	−0.034	0.229	0.343	0.229	0.943	1.149
100	0.20	0.02	−0.090	0.370	−0.058	0.267	0.308	0.267	0.951	1.164
100	0.20	0.02	−0.076	0.395	−0.043	0.273	0.298	0.273	0.940	1.103
100	0.20	0.05	−0.060	0.377	−0.038	0.271	0.315	0.271	0.948	1.171
100	0.20	0.05	−0.073	0.362	−0.043	0.249	0.297	0.249	0.956	1.120
100	0.20	0.10	−0.059	0.398	−0.036	0.287	0.319	0.287	0.950	1.190
100	0.20	0.10	−0.062	0.392	−0.035	0.271	0.297	0.271	0.951	1.117
100	0.40	0.02	−0.145	0.335	−0.053	0.155	0.169	0.155	0.955	0.661
100	0.40	0.02	−0.195	0.361	−0.055	0.149	0.158	0.149	0.939	0.617
100	0.40	0.05	−0.158	0.367	−0.058	0.169	0.170	0.169	0.933	0.666
100	0.40	0.05	−0.202	0.366	−0.057	0.151	0.160	0.152	0.944	0.625
100	0.40	0.10	−0.139	0.363	−0.049	0.167	0.171	0.166	0.952	0.672
100	0.40	0.10	−0.197	0.372	−0.057	0.151	0.160	0.151	0.948	0.627

[†] Mean, SE: Mean and standard deviation of the estimates, $\widehat{SE}$: Mean of the standard error estimates, RMSE: Root mean squared error of the estimates, CR, EW: Coverage rate and expected width of the 95% confidence intervals.

e-Table 8. Results of the simulation studies for the regression coefficient estimators of β_4 (= −0.05; N = 200, 500)[†].

			Linear mixed-effects model (log odds ratio)		Pseudo-observations approach (log risk ratio)					
N	P_{event}	τ	Mean	SE	Mean	SE	$\widehat{SE}$	RMSE	CR	EW
200	0.20	0.02	−0.035	0.263	−0.021	0.190	0.193	0.192	0.937	0.757
200	0.20	0.02	−0.062	0.251	−0.039	0.175	0.184	0.175	0.947	0.722
200	0.20	0.05	−0.068	0.270	−0.044	0.193	0.194	0.192	0.950	0.762
200	0.20	0.05	−0.063	0.276	−0.037	0.191	0.185	0.191	0.930	0.727
200	0.20	0.10	−0.061	0.264	−0.040	0.188	0.194	0.188	0.953	0.761
200	0.20	0.10	−0.087	0.258	−0.054	0.177	0.184	0.177	0.943	0.720
200	0.40	0.02	−0.136	0.239	−0.050	0.112	0.113	0.112	0.937	0.442
200	0.40	0.02	−0.184	0.248	−0.050	0.101	0.104	0.101	0.945	0.406
200	0.40	0.05	−0.127	0.237	−0.044	0.111	0.113	0.111	0.955	0.442
200	0.40	0.05	−0.184	0.254	−0.051	0.104	0.104	0.104	0.941	0.407
200	0.40	0.10	−0.135	0.245	−0.047	0.112	0.113	0.112	0.946	0.444
200	0.40	0.10	−0.180	0.249	−0.049	0.101	0.105	0.101	0.950	0.411
500	0.20	0.02	−0.063	0.160	−0.043	0.115	0.118	0.115	0.947	0.461
500	0.20	0.02	−0.074	0.160	−0.047	0.110	0.112	0.110	0.944	0.439
500	0.20	0.05	−0.074	0.171	−0.049	0.123	0.118	0.123	0.932	0.462
500	0.20	0.05	−0.079	0.162	−0.048	0.112	0.112	0.111	0.956	0.436
500	0.20	0.10	−0.064	0.162	−0.043	0.116	0.118	0.116	0.955	0.461
500	0.20	0.10	−0.075	0.165	−0.046	0.113	0.112	0.113	0.945	0.439
500	0.40	0.02	−0.124	0.146	−0.045	0.067	0.069	0.067	0.951	0.269
500	0.40	0.02	−0.185	0.161	−0.053	0.067	0.064	0.067	0.935	0.249
500	0.40	0.05	−0.130	0.146	−0.047	0.067	0.069	0.067	0.958	0.270
500	0.40	0.05	−0.179	0.158	−0.051	0.065	0.064	0.065	0.944	0.249
500	0.40	0.10	−0.134	0.157	−0.048	0.072	0.069	0.072	0.936	0.271
500	0.40	0.10	−0.175	0.160	−0.048	0.066	0.064	0.066	0.941	0.252

[†] Mean, SE: Mean and standard deviation of the estimates, $\widehat{SE}$: Mean of the standard error estimates, RMSE: Root mean squared error of the estimates, CR, EW: Coverage rate and expected width of the 95% confidence intervals.

e-Table 9. Results of the simulation studies for the regression coefficient estimators of β_5 (= −0.01; N = 50, 100) †.

			Linear mixed-effects model (log odds ratio)		Pseudo-observations approach (log risk ratio)					
N	P_{event}	τ	Mean	SE	Mean	SE	$\widehat{SE}$	RMSE	CR	EW
50	0.20	0.02	−0.014	0.018	−0.010	0.013	0.015	0.013	0.940	0.058
50	0.20	0.02	−0.013	0.018	−0.009	0.013	0.014	0.013	0.941	0.056
50	0.20	0.05	−0.012	0.018	−0.008	0.013	0.014	0.013	0.939	0.056
50	0.20	0.05	−0.013	0.017	−0.009	0.012	0.014	0.012	0.941	0.056
50	0.20	0.10	−0.013	0.019	−0.009	0.014	0.015	0.014	0.942	0.058
50	0.20	0.10	−0.013	0.018	−0.009	0.013	0.014	0.013	0.941	0.057
50	0.40	0.02	−0.021	0.015	−0.009	0.007	0.008	0.007	0.944	0.032
50	0.40	0.02	−0.024	0.017	−0.009	0.008	0.008	0.008	0.934	0.032
50	0.40	0.05	−0.020	0.016	−0.008	0.007	0.008	0.007	0.947	0.032
50	0.40	0.05	−0.023	0.016	−0.008	0.007	0.008	0.007	0.953	0.031
50	0.40	0.10	−0.021	0.016	−0.008	0.007	0.008	0.007	0.954	0.032
50	0.40	0.10	−0.022	0.017	−0.008	0.007	0.008	0.007	0.943	0.032
100	0.20	0.02	−0.012	0.011	−0.009	0.008	0.009	0.008	0.946	0.034
100	0.20	0.02	−0.012	0.011	−0.008	0.008	0.008	0.008	0.943	0.033
100	0.20	0.05	−0.012	0.011	−0.008	0.008	0.009	0.008	0.945	0.034
100	0.20	0.05	−0.012	0.012	−0.008	0.009	0.009	0.009	0.927	0.034
100	0.20	0.10	−0.012	0.012	−0.008	0.009	0.009	0.009	0.936	0.035
100	0.20	0.10	−0.012	0.012	−0.008	0.008	0.008	0.008	0.926	0.033
100	0.40	0.02	−0.020	0.010	−0.008	0.005	0.005	0.005	0.941	0.020
100	0.40	0.02	−0.022	0.011	−0.008	0.005	0.005	0.005	0.959	0.019
100	0.40	0.05	−0.020	0.011	−0.008	0.005	0.005	0.005	0.927	0.020
100	0.40	0.05	−0.022	0.011	−0.008	0.005	0.005	0.005	0.932	0.019
100	0.40	0.10	−0.020	0.010	−0.008	0.005	0.005	0.005	0.944	0.020
100	0.40	0.10	−0.021	0.011	−0.008	0.005	0.005	0.005	0.950	0.020

† Mean, SE: Mean and standard deviation of the estimates, $\widehat{SE}$: Mean of the standard error estimates, RMSE: Root mean squared error of the estimates, CR, EW: Coverage rate and expected width of the 95% confidence intervals.

e-Table 10. Results of the simulation studies for the regression coefficient estimators of β_5 (= −0.01; N = 200, 500) [†].

			Linear mixed-effects model (log odds ratio)		Pseudo-observations approach (log risk ratio)					
N	P_{event}	τ	Mean	SE	Mean	SE	$\widehat{SE}$	RMSE	CR	EW
200	0.20	0.02	−0.012	0.008	−0.008	0.006	0.006	0.006	0.939	0.022
200	0.20	0.02	−0.012	0.008	−0.008	0.006	0.006	0.006	0.943	0.022
200	0.20	0.05	−0.012	0.008	−0.008	0.006	0.006	0.006	0.952	0.023
200	0.20	0.05	−0.012	0.008	−0.008	0.006	0.006	0.006	0.938	0.022
200	0.20	0.10	−0.011	0.008	−0.008	0.006	0.006	0.006	0.943	0.022
200	0.20	0.10	−0.012	0.008	−0.008	0.006	0.006	0.006	0.945	0.022
200	0.40	0.02	−0.020	0.007	−0.008	0.003	0.003	0.003	0.954	0.013
200	0.40	0.02	−0.022	0.008	−0.008	0.003	0.003	0.003	0.937	0.013
200	0.40	0.05	−0.020	0.007	−0.008	0.003	0.003	0.003	0.949	0.013
200	0.40	0.05	−0.021	0.007	−0.008	0.003	0.003	0.003	0.950	0.013
200	0.40	0.10	−0.019	0.007	−0.008	0.003	0.003	0.003	0.936	0.014
200	0.40	0.10	−0.021	0.007	−0.008	0.003	0.003	0.003	0.954	0.013
500	0.20	0.02	−0.011	0.005	−0.008	0.004	0.004	0.004	0.947	0.014
500	0.20	0.02	−0.012	0.005	−0.008	0.004	0.003	0.004	0.930	0.014
500	0.20	0.05	−0.011	0.005	−0.008	0.004	0.004	0.004	0.947	0.014
500	0.20	0.05	−0.012	0.005	−0.008	0.004	0.003	0.004	0.936	0.014
500	0.20	0.10	−0.011	0.005	−0.008	0.004	0.004	0.004	0.951	0.014
500	0.20	0.10	−0.012	0.005	−0.008	0.004	0.003	0.004	0.948	0.014
500	0.40	0.02	−0.019	0.005	−0.008	0.002	0.002	0.002	0.925	0.008
500	0.40	0.02	−0.022	0.005	−0.008	0.002	0.002	0.002	0.944	0.008
500	0.40	0.05	−0.020	0.004	−0.008	0.002	0.002	0.002	0.946	0.008
500	0.40	0.05	−0.021	0.005	−0.008	0.002	0.002	0.002	0.943	0.008
500	0.40	0.10	−0.019	0.004	−0.008	0.002	0.002	0.002	0.951	0.008
500	0.40	0.10	−0.021	0.005	−0.007	0.002	0.002	0.002	0.935	0.008

[†] Mean, SE: Mean and standard deviation of the estimates, $\widehat{SE}$: Mean of the standard error estimates, RMSE: Root mean squared error of the estimates, CR, EW: Coverage rate and expected width of the 95% confidence intervals.

e-Appendix B: Additional simulations for frequent and rare event data

We conducted additional simulation studies to assess the performances of the inference methods under frequent and rare event situations. We considered the event rates at 10%, 60%, and 80%. We also set the sample size N at 100 and 200, the standard deviation τ of the random-effects distribution at 0.05 and 0.10, and the log risk ratio β_1 at 0.10 (to ensure the event rates of both groups do not exceed 100%; especially for the scenarios that the event rates are 80%) in these supplementary simulations. The simulation results for β_1 are presented in e-Table 11. Note that we attempted simulation studies under more rare event settings (e.g., 5%), the ordinary logistic mixed-effects model analysis provided irregular estimates (e.g., extremely large estimates) frequently, and we could not conduct valid evaluations. These situations correspond to the well-known "separation" or "quasi-separation" conditions (Albert and Anderson, 1984; Uno, Noma and Gosho, 2024), and these methods would have substantial difficulties under these conditions. Development of advanced methods to address this problem (e.g., Firth-type correction; Firth, 1993) would also be a relevant problem in future studies. However, when the sample size N gets larger, these methods would also be effective. Overall, the simulation results in e-Table 11 consistently demonstrated favorable performance of the proposed methods.

e-Table 11. Results of the simulation studies for the regression coefficient estimators of β_1 (= 0.10)[†].

			Linear mixed-effects model (log odds ratio)		Pseudo-observations approach (log risk ratio)					
N	P_{event}	τ	Mean	SE	Mean	SE	$\widehat{SE}$	RMSE	CR	EW
100	0.10	0.05	0.128	0.373	0.113	0.331	0.365	0.332	0.937	1.370
100	0.10	0.10	0.107	0.378	0.095	0.336	0.342	0.335	0.924	1.280
200	0.10	0.05	0.119	0.268	0.105	0.238	0.229	0.238	0.929	0.895
200	0.10	0.10	0.120	0.251	0.107	0.223	0.227	0.223	0.948	0.889
100	0.60	0.05	0.261	0.223	0.102	0.087	0.088	0.087	0.948	0.344
100	0.60	0.10	0.258	0.230	0.100	0.089	0.089	0.089	0.944	0.347
200	0.60	0.05	0.246	0.160	0.097	0.063	0.062	0.063	0.942	0.240
200	0.60	0.10	0.251	0.161	0.097	0.063	0.062	0.063	0.942	0.243
100	0.80	0.05	0.539	0.285	0.101	0.053	0.053	0.053	0.950	0.209
100	0.80	0.10	0.532	0.300	0.097	0.054	0.055	0.054	0.941	0.215
200	0.80	0.05	0.529	0.199	0.100	0.038	0.038	0.037	0.951	0.147
200	0.80	0.10	0.540	0.207	0.099	0.038	0.039	0.038	0.953	0.152

[†] Mean, SE: Mean and standard deviation of the estimates, $\widehat{SE}$: Mean of the standard error estimates, RMSE: Root mean squared error of the estimates, CR, EW: Coverage rate and expected width of the 95% confidence intervals.

e-Appendix C: Computational times of the simulation studies

As supplementary data, we provide computational times for the total 48 scenarios of simulation studies in Section 4 in e-Tables 12 and 13. Note that we used several computers that adopt different CPUs (Intel Core i9 14900KS and Intel Xeon Gold 6338) to implement the large simulation studies, and the absolute computational times between the different CPU machines are not comparable; please use them as reference information. The computational times of the proposed pseudo-observations approach are relatively large, but they can be dramatically reduced if parallel computation tools are used (see the example R codes in Supporting Information materials).

e-Table 12. Mean computational times for the simulation studies (N = 50, 100)*.

N	P_{event}	τ	β_1	Linear mixed-effects model	Pseudo-observations approach †
50	0.20	0.02	0.47	0.11 seconds	2.47 minutes
50	0.20	0.02	0.69	0.11 seconds	2.48 minutes
50	0.20	0.05	0.47	0.11 seconds	2.46 minutes
50	0.20	0.05	0.69	0.11 seconds	2.48 minutes
50	0.20	0.10	0.47	0.11 seconds	2.47 minutes
50	0.20	0.10	0.69	0.11 seconds	2.48 minutes
50	0.40	0.02	0.47	0.11 seconds	2.56 minutes
50	0.40	0.02	0.69	0.11 seconds	2.58 minutes
50	0.40	0.05	0.47	0.11 seconds	2.55 minutes
50	0.40	0.05	0.69	0.11 seconds	2.58 minutes
50	0.40	0.10	0.47	0.11 seconds	2.55 minutes
50	0.40	0.10	0.69	0.12 seconds	2.57 minutes
100	0.20	0.02	0.47	0.23 seconds	4.76 minutes
100	0.20	0.02	0.69	0.24 seconds	4.78 minutes
100	0.20	0.05	0.47	0.23 seconds	4.76 minutes
100	0.20	0.05	0.69	0.24 seconds	4.78 minutes
100	0.20	0.10	0.47	0.24 seconds	4.76 minutes
100	0.20	0.10	0.69	0.24 seconds	4.76 minutes
100	0.40	0.02	0.47	0.20 seconds	4.97 minutes
100	0.40	0.02	0.69	0.21 seconds	5.03 minutes
100	0.40	0.05	0.47	0.20 seconds	4.98 minutes
100	0.40	0.05	0.69	0.22 seconds	5.05 minutes
100	0.40	0.10	0.47	0.20 seconds	5.00 minutes
100	0.40	0.10	0.69	0.21 seconds	5.06 minutes

† Per single core computation. The computational times can be reduced by using parallel computations.
* Workstations with Intel Core i9 14900KS CPU are used for the 24 scenarios.

e-Table 13. Mean computational times for the simulation studies (N = 200, 500) *.

N	P_{event}	τ	β_1	Linear mixed-effects model	Pseudo-observations approach †
200	0.20	0.02	0.47	0.71 seconds	15.72 minutes
200	0.20	0.02	0.69	0.71 seconds	15.80 minutes
200	0.20	0.05	0.47	0.71 seconds	15.73 minutes
200	0.20	0.05	0.69	0.72 seconds	15.81 minutes
200	0.20	0.10	0.47	0.72 seconds	15.73 minutes
200	0.20	0.10	0.69	0.71 seconds	15.78 minutes
200	0.40	0.02	0.47	0.74 seconds	16.05 minutes
200	0.40	0.02	0.69	0.78 seconds	16.12 minutes
200	0.40	0.05	0.47	0.74 seconds	16.05 minutes
200	0.40	0.05	0.69	0.78 seconds	16.09 minutes
200	0.40	0.10	0.47	0.74 seconds	16.04 minutes
200	0.40	0.10	0.69	0.78 seconds	16.11 minutes
500	0.20	0.02	0.47	1.86 seconds	39.62 minutes
500	0.20	0.02	0.69	1.92 seconds	39.60 minutes
500	0.20	0.05	0.47	1.86 seconds	39.62 minutes
500	0.20	0.05	0.69	1.91 seconds	39.56 minutes
500	0.20	0.10	0.47	1.85 seconds	39.65 minutes
500	0.20	0.10	0.69	1.90 seconds	39.61 minutes
500	0.40	0.02	0.47	1.61 seconds	41.83 minutes
500	0.40	0.02	0.69	1.61 seconds	42.01 minutes
500	0.40	0.05	0.47	1.63 seconds	41.97 minutes
500	0.40	0.05	0.69	1.59 seconds	41.93 minutes
500	0.40	0.10	0.47	1.60 seconds	42.02 minutes
500	0.40	0.10	0.69	1.62 seconds	42.11 minutes

† Per single core computation. The computational times can be reduced by using parallel computations.
* A workstation with Intel Xeon Gold 6338 CPU is used for the 24 scenarios.

References

Albert, A., and Anderson, J. A. (1984). On the existence of the maximum likelihood estimates in logistic regression models. *Biometrika* **71**, 1-10.

Firth, D. (1993). Bias reduction of maximum likelihood estimates. *Biometrika* **80**, 27-38.

Uno, S., Noma, H., and Gosho, M. (2024). Firth-type penalized methods of the modified Poisson and least-squares regression analyses for binary outcomes. *Biom J* **66**, e202400004.
